# Resistance to Clopidogrel among Iranian Patients Undergoing Angioplasty Intervention 

**Published:** 2013

**Authors:** Mohammad Haji Aghajani, Farzad Kobarfard, Olia Safi, Kourosh Sheibani, Mohammad Sistanizad

**Affiliations:** a *Department of Cardiology, Imam Hussein Medical and Educational Center, Shahid Beheshti University of Medical Sciences, Tehran, Iran.*; b *Department of Medical Chemistry, School of Pharmacy, Shahid Beheshti University of Medical Sciences, Tehran, Iran. *; c *Department of Clinical Pharmacy, School of Pharmacy, Shahid Beheshti University of Medical Sciences, Tehran, Iran. *; d *Clinical Research and Development Center, Imam Hussein Medical Center, Shahid Beheshti University of Medical Sciences, Tehran, Iran. *; e *Department of Pharmaceutical Care, Imam Hussein Medical and Educational Center, Shahid Beheshti University of Medical Sciences, Tehran, Iran. *

**Keywords:** Coronary, Angioplasty, Clopidogrel, Drug resistance

## Abstract

To study the resistance to standard dosage of clopidogrel among Iranian patients following percutaneous coronary intervention measured by platelet aggregation test.

Patients undergoing percutaneous coronary intervention in Imam Hussein Medical center, Tehran, Iran, who were under treatment with aspirin, but had no history of clopidogrel usage, entered the study. Patients received standard dosage of clopidogrel (Plavix^®^, Sanofi, France, 600 mg loading dose and 75 mg/day afterward). Platelet aggregation was measured using light transmission aggregometer. The response to the drug was categorized as complete resistance (platelet aggregation decreased less than 10%), intermediate resistance (platelet aggregation decreased between 10 to 30%) and complete response (platelet aggregation decreased to 30% or more). All patients were evaluated for major adverse cardio vascular events one month after the angioplasty based on MACE criteria by phone contact.

Thirty-one patients with a mean age of 59 ± 13 entered the study. Sixty-five percent of patients showed complete response to clopidogrel (95% CI: 45% to 81%), 22% showed intermediate resistance (95% CI: 10-41%) and 13% showed complete resistance (95% CI: 4-30%). One month after the angioplasty, no major adverse cardiovascular event was recorded.

Based on our findings, it seems that there is no major difference between Iranian population and other studies regarding the resistance to clopidogrel. Due to the limited number of participants in our study, further investigations with higher number of patients are recommended to more precisely calculate the percentage of resistance among Iranian patients.

## Introduction

Cardiovascular diseases are becoming the main cause of mortality and morbidity throughout the world. The high prevalence of this disease has resulted in the introduction of more advanced treatment methods and drugs in recent decades (1). One of the most dangerous cardiac diseases in coronary arteries occlusion, which in some cases results in the need for surgical procedures, in the form of angioplasty or bypass surgery. In last 30 years, newer methods like stent implantation and new drug regiments have resulted in better outcomes (2). The invention of these new methods has caused some new concerns (1). Placing a stent is one of the methods used to treat the occlusion which is performed using a bare metal or a drug-eluting stent, both of which can result in thrombosis (3).

Platelets play a critical role in acute coronary syndrome (ACS) caused by thrombotic events during and after the percutaneous coronary intervention (PCI). Aspirin and clopidogrel are prophylactic drugs used in patients undergoing angioplasty to reduce the rate of thrombosis and related morbidity and mortality (3). Clopidogrel is a pro-drug which is activated by CYP450 liver enzyme (4).

The active metabolite of clopidogrel makes an irreversible bond with P2Y12 ADP receptor and stops the platelet aggregation (5). Eighty-five percent of clopidogrel is hydrolyzed to inactive carboxylic acid derivatives and 15% is oxidized into 2-oxyclopidogrel by CY450 and then turned into active thiol derivatives (6), with CYP2C19 being involved in both pathways (7).

Clopidogrel is a selective ADP-receptor antagonist that bonds to this receptor in platelet surface as a non-competitive antagonist and stops the ADP from reaching its receptor. This results in a reduction in activity of glycoprotein in platelet (GPIIb/IIIa) needed for fibrinogen-platelet adhesion (8).

The antiplatelet activity of clopidogrel is not equal in all patients. Some patients do not show a good response to clopidogrel (3) and have a higher risk of thrombosis. It has been demonstrated that this resistance is related to higher rate of cardiovascular events in these group of patients compared to nonresistant patients (3). Between 4 to 30 percent of patients treated with 75 mg/day of clopidogrel has been reported to show resistance in different studies (3). Interpersonal difference in response to clopidogrel is multifactorial and can be due to intrinsic or extrinsic mechanisms (3).

Extrinsic mechanisms can be related to low dosage in patients with a history of PCI or interference with other drugs and intrinsic factors are gene polymorphism or up regulation of other platelet activity pathways. In previous studies, clopidogrel non-responders have been defined as those patients whose platelet activity after drug usage drops less than 10% compared to their level before using drug. Patients whose platelet activity drops between 10 to 30% after the drug, are referred as semi-responder and complete response is defined as more than 30% reduction in activity compared to pre-drug state (3).

To the best of our knowledge, the prevalence of drug resistance to clopidogrel and its role in therapeutic outcome in Iran have not been investigated. The present study was performed to access the prevalence of drug resistance and the role of this resistance in patients’ outcomes among Iranian patients and methods.

## Experimental

*Patients and methods*

The population in the present study was comprised of patients undergoing angioplasty with a stent placement in the department of cardiology, Imam Hossein Medical Center, Tehran, Iran. All patients were given a written informed consent before entering the study, and the study was performed in accordance with the declaration of Helsinki.

Inclusion criteria were angioplasty procedure with drug elating stent (DES) placement and consuming standard dosage of aspirin (75-325mg/day). The exclusion criteria were age under 18, history of clopidogrel usage in last 1 month, acute infarction in last 18 h, platelet count < 100000 mm3, hematocrit < 25%, creatinine > 4 mg/d, usage of glycoprotein IIb/IIIa before procedure, history of alcohol consumption, previous PCI, contraindications due to any cause for anticoagulant therapy, severe disease with life expectancy under 1 year and finally patients with cancer or patients undergoing dialysis. Blood sampling from patients was performed from June 2011 to June 2012. For all patients entering the study, a questionnaire including demographic information was completed.

After entering the study, patients received a loading dose of 600 mg clopidogrel (Plavix^®^, Sanofi, France) before the angioplasty and a daily dose of 75 mg afterward. Platelet aggregation test was performed for all patients before and 24 h after taking the loading dose of clopidogrel using light transmission aggregometry (LTA) method using a 4-channel Labtec APACT 4004 aggregometer. LTA is the gold standard method for studying the platelet activity (2). The response to drug was categorized as complete resistance (platelet aggregation decreased less than 10%), intermediate resistance (platelet aggregation decreased between 10% to 20%) and complete response (platelet aggregation decreased 30% or more). All patients were interviewed by phone 1 month after the angioplasty regarding MACE criteria including deaths, nonfatal MI, and need for urgent revascularization.

## Results

Thirty-one patients with an average age of 59 ± 13 entered the study. Twelve patients were female and 19 were male. Demographic findings of patients are summarized in Table 1.

**Table 1:** Demographic findings of 31 patients entering the study

**Parameter**	**Value**		**Parameter**	**Value**
Age	59 ± 13		Pantoprazol	3 (9.7%)
	59 (42 to 81)		Atorvastatin	18 (58.1%)
Gender	F	12 (38.7%)
	M	19 (61.3%)
Major Risk Factors				
Diabetes	6 (19.4%)		Beta Blockers	18 (58.1%)
HTN	17 (54.8%)		Nitrates	12 (38.7%)
HLP	3 (9.7%)		Omeprazol	2 (6.5%)
Smoking	7 (22.6%)			

Results are presented as mean ± SD, median (range) and frequency (%)

The average platelet aggregation among patients before and after the drug administration is demonstrated in Table 2.

**Table 2:** Platelet aggregation among patients before and after therapy

**Statistics**	**Pre**	**Post**	**Change**	**Relative change (%)**
Mean SD	54.5 ± 18.8	30.4 ± 19.8	24.1 ± 20.1	41 ± 36
Median (Range)	54.7 (14.8 to 88.6)	22.5 (3.4 to 76.7)	23.7 (-7.3 to 60.4)	40 (-28 to 94)
95% CI	47.6 to 61.4	23.1 to 37.6	16.8 to 31.5	27.9 to 54.3

95%                    CI:                      95%                     Confidence                       Interval

P for change < 0.001 based on paired t-test.

This was 54.2 ± 188 before and was reduced to 30.4 ± 19.8 after the therapy which shows a reduction of 24.1 ± 20.1. The percentage of patients who were complete responder, semi-responder or non-responder is shown in Table 3.

**Table 3:** Percentage of complete, semi and non-responders to clopidogrel among patients

**Response**	**N**	**%**	95% CI for percent*
Complete Responder	20	65%	45% to 81%
Semi Responder	7	22%	10% to 41%
Non-responder	4	13%	4% to 30%

* Based on Exact method

**Figure 1:**
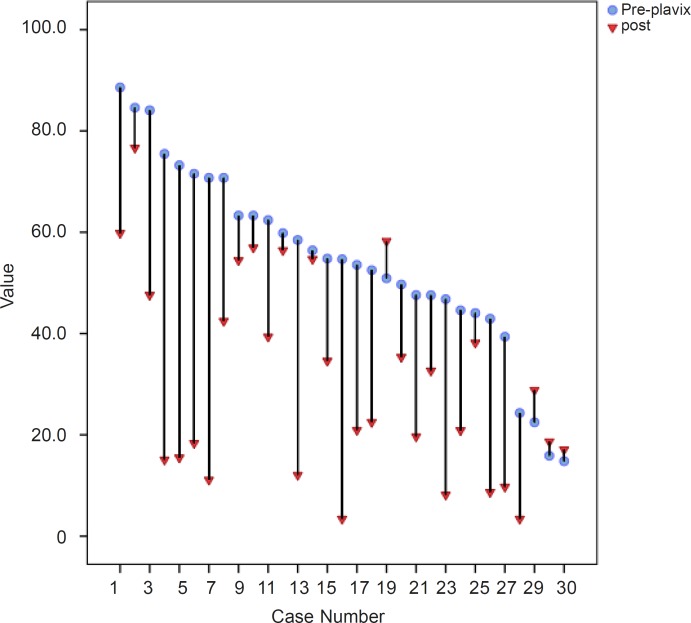
Reduction of platelet aggregation for all patients

Based on our findings, 65% of patents had complete response and 22% showed semi-resistance and 13% showed complete resistance to drug. The reduction of platelet aggregation for all patients demonstrated in Figure 1.

## Discussion

The percentage of drug response to clopidogrel in different populations has been reported in several studies. Lau *et al. *reported 22 percent resistance and 22% semi-resistance after 300 mg loading and 75 mg daily dose of clopidogrel in patients from USA which is somehow higher than our findings and might be related to lower loading dose in their study(9). They suggested the interpersonal differences in CYP3A4 activity among patients as the cause of this drug response variation (9). Lapantalo *et al. *used a loading dose of 600 mg and daily dose of 75 mg among Finnish patients and reported 20% resistance among 1608 patients, which is in line with our findings (10). In another study, Schulz *et al. *in Germany reported 20% resistance in patients undergoing angioplasty and stent placement (11). Geisler *el al. *reported a 5.8% resistance among German patients which is less than our findings (3).

In a study by Neubauer *et al*., using combination therapy with aspirin and clopidogrel among 504 patients, it was found that 30.8% of patients are resistant. Resistance to clopidogrel might be due to patient’s clinical condition or genetic factors (12). They proposed that a less active ADP receptor among some patients might cause the clopidogrel to have a somehow muted effect as ADP receptor antagonist (12). They suggested an increase of daily dose from 75 mg to 150 mg. The results of some several studies are summarized in Table 4.

**Table 4:** Results of some previous studies regarding resistance to clopidogrel

**Study**	**N**	**Patients**	**Dose (mg, load/qd)**	**Prevalence%**
Gurbel et al. (13)	92	PCI	300/75	31-35
Jaremo et al. (14)	18	PCI	300/75	28
Muller et al. (15)	119	PCI	600/75	5-11
Mobley et al. (16)	50	PCI	300/75	30
Lepantalo et al. (10)	50	PCI	300/75	40
Angiolillo et al. (17)	48	PCI	300/75	44
Matetzky et al. (18)	60	STEMI	300/75	25
Dziewierz et al. (19)	31	CAD	300	23
Lev et al. (20)	150	PCI	300	24
Angiolillo et al. (21)	52	Diabetics and Non-diabetics	300	38 (diabetic) 8 (Non-diabetic)
Gurbel et al. (22)	190	PCI	300 or 600/75	28-32 (300 mg) 8 (600 mg)

These findings by different authors and on different populations show a variation regarding resistance to clopidogrel. Our study shows that the resistance is present among our patients but it seems that the prevalence of resistance is in line with the majority of previous studies. This study was among first studies on the resistance to clopidogrel in Iranian patients and had some limitations. The study population was limited to patients undergoing elective PCI and no emergency patient entered the study. In addition, we studied only patients who received a stent implantation during the angioplasty. Finally, our sample size of 31 patients causes our percentages regarding the prevalence of resistance to have a wide range and moderate reliability. Considering these limitations, we suggest bigger multicentric studies to cover a wider group of patients and calculate more precisely the prevalence of resistance to clopidogrel among Iranian patients.

## Conclusion

Based on our findings, it seems that there is no major difference between Iranian population and other studies regarding the resistance to clopidogrel. Due to the limited number of participants in our study, further investigations with higher number of patients is recommended to more precisely calculate the percentage of resistance among the Iranian patients.
